# Statins in the Cause and Prevention of Cancer: Confounding by Indication and Mediation by Rhabdomyolysis and Phosphate Toxicity

**DOI:** 10.3390/jcdd11090296

**Published:** 2024-09-23

**Authors:** Ronald B. Brown

**Affiliations:** School of Public Health Sciences, University of Waterloo, Waterloo, ON N2L 3G1, Canada; r26brown@uwaterloo.ca

**Keywords:** statin, atherosclerosis, cancer, hypercholesterolemia, hydroxymethylglutaryl-coenzyme A reductase inhibitor, oxysterol, phosphate toxicity, rhabdomyolysis, tumorigenesis

## Abstract

Statins are drugs used in cardiovascular pharmacotherapy to decrease hypercholesterolemia and lower the risk of atherosclerosis. Statins also increase the risk of rhabdomyolysis, which is often minimized in comparison with large relative risk reductions of cardiovascular disease reported in clinical trials. By contrast, absolute risk reductions of cardiovascular disease are often clinically insignificant and unreported in statin clinical trials. Additionally, cytotoxic effects of statins inhibit cancer cell proliferation and reduce cancer risk, but other studies found that statins are carcinogenic. Due to an inverse association between incidence of cancer and atherosclerosis, the indication to prescribe statins likely biases the association of statins with cancer prevention. Dietary patterns associated with atherosclerosis and cancer contain inverse amounts of cholesterol and phosphate, an essential mineral that stimulates tumorigenesis. Accordingly, lower cancer risk is associated with high dietary cholesterol intake and increased risk of atherosclerosis. Furthermore, serum is exposed to excessive inorganic phosphate that could increase cancer risk as rhabdomyolysis induced by statins releases phosphate from skeletal muscle breakdown. Increased risk of comorbid conditions associated with statins may share the mediating factor of phosphate toxicity. More research is warranted on statins in the cause and prevention of cancer.

## 1. Introduction

Statins are cardiovascular pharmacotherapy drugs that inhibit 3-hydroxy-3-methyl-glutaryl-coenzyme A (HMG-Co-A) reductase, and are used by more than 200 million people around the world to reduce serum levels of low-density lipoprotein cholesterol (LDL-C) [1]. A study of 91 countries found that statin use among adults 40 years and older increased by 24.7% between 2015 and 2020 [2]. Statin therapy was originally intended to reduce hypercholesterolemia and prevent atherosclerosis and coronary heart disease by inhibiting cholesterol biosynthesis. But statins have also been repurposed (new uses for old drugs) to reduce cancer cell proliferation, induce apoptosis, and increase chemotherapy efficacy [3,4]. For example, the mevalonate signaling pathway, a rate-limiting step in cholesterol biosynthesis, contributes to tumorigenesis by increasing critical enzymes and transcription factors, and statins can block the mevalonate pathway to target cancer cells with antitumor effects [5].

Jiang et al. noted that anticancer properties of statins have been reported in many epidemiological and clinical studies, although the findings are not consistent [6]. Examples of studies finding an anticancer effect of statins include the following: Nielsen et al. found that statins used by cancer patients were associated with reduced cancer-related mortality in 13 different types of cancer compared to cancer patients who never used statins [7]. The authors suggested that limited proliferation of cancer cells may be related to a reduction in available cholesterol. Mei et al. conducted a systematic review and meta-analysis of over one-million individuals from 95 cohorts and found that statin use was significantly associated with reduced cancer-specific mortality and reduced cancer progression compared to no statin use [8]. The authors suggested that the evidence supports the use of statin therapy as an adjuvant therapy for cancer treatment. Tamburrino et al. conducted a systematic review and meta-analysis and found improved survival associated with statin use among over 33,000 patients with pancreatic ductal adenocarcinoma [9]. Majidi et al. used the Ovarian Cancer Prognosis and Lifestyle prospective observational study to examine statin use by Australian women with ovarian cancer [10]. The researchers found that statin therapy was associated with improved survival following ovarian cancer diagnosis. 

Furthermore, Islam et al. examined risk of hepatocellular carcinoma using a systematic review and meta-analysis [11]. Among over 59,000 study participants from four continents, the study found that statin use was associated with a lower risk of hepatocellular carcinoma compared to no statin use. Voorneveld et al. analyzed Netherlands data on use of statins in 999 patients after a diagnosis of colon cancer [12]. Statin use in the study was significantly associated with reduced all-cause mortality and reduced cancer mortality. El-Serag et al. found that statin use was significantly associated with reduced risk of hepatocellular carcinoma in a nested case-control study of cohort participants with diabetes [13]. Beckwitt et al. found that statins were associated with weakened metastatic spread of breast cancer to the liver and lungs [14], and Liu et al. conducted a meta-analysis of 42 studies and found that statin use was associated with reduced risk of colorectal cancer [15]. 

Yet, other studies failed to find an anticancer effect from statins, including the following: Bonovas et al. did not find a statistically significant protective effect of statin use associated with risk of breast cancer in a meta-analysis [16]. Konings et al. found no benefits from adding pravastatin to treatments for advanced gastric cancer [17]. Lee et al. tested the addition of simvastatin to treatment for nonadenocarcinomatous non-small cell lung cancer and found no clinical benefits [18]. Eliassen et al. analyzed over 3100 cases of breast cancer incidence in the Nurses’ Health Study and found similar breast cancer risk among patients using statins compared to non-users [19]. Islam et al. conducted a more recent meta-analysis in 2017 and found no association between breast cancer risk and statin use [20]. McMenamin et al. also found little protection against cancer-specific mortality associated with statin use in a national cohort of breast cancer patients in Ireland [21].

Moreover, other studies investigating the anticancer effects of statins found a mix of both positive and negative results in the same study, including weak and/or statistically insignificant anticancer findings. In 2018, Abdullah et al. recommended design improvements of prospective clinical trials to better address the lack of consensus regarding efficacy of statins for oncotherapies [22]. The researchers also suggested that inadequate control of confounding factors may have affected cancer trial outcomes, such as the health status of patients who use statins compared to non-users. In addition to anticancer effects, carcinogenic effects from statins have also been reported [23], which may not be unexpected considering Blagosklonny’s observation that cancer can be treated with the same toxic agent that causes cancer [24]. However, mechanisms explaining statins’ contradictory relationship in causing and preventing cancer require further investigations. The aim of this review is to examine the research literature and propose plausible mechanisms by which statin use is associated with both increased and decreased cancer risk.

## 2. Materials and Methods

The present paper used a grounded theory literature-review method published by Wolfswinkel et al. [25] to collect and analyze a wide scope of evidence associating statins with the cause and prevention of cancer. Sociologists Glaser and Strauss originally developed grounded theory as a method to acquire in-depth understanding of a specific research area [26]. Grounded theory adds rigor and objectivity to research by removing all preconceived assumptions about the research problem and uses an inductive approach to gradually construct a new theory based exclusively on the collected evidence. Additionally, the method by Wolfswinkel et al. allows the researcher to focus grounded theory on research findings across a broad range of areas. This helps the researcher discover new interconnections of knowledge, acquire new insights and perspectives on the research problem, fill in gaps in the research literature, and uncover new directions for further interdisciplinary research. 

All relevant literature sources were considered in the selection of studies for the present review, regardless of study design or date. Concepts were identified through rigorous comparative analysis of selected study findings, and themes were synthesized in an iterative process of induction until an explanatory theory emerged. The paper’s narrative style presents an evidence-based proposal that phosphate toxicity, the harmful accumulation of dysregulated inorganic phosphate in body tissues [27], is a mediating factor in the controversial association of statins with the cause of cancer. Furthermore, confounding by indication is proposed to account for statins’ association with cancer prevention, which is also potentially mediated by reduced phosphate toxicity.

## 3. Risk Calculations in Statin Trials

The low risk of adverse effects from statins is often minimized when compared to the much larger benefits claimed for statins in reducing cardiovascular risks [28], but more recent findings challenge this unbalanced risk-benefits assessment. In 2022, the Journal of the American Medical Association published two meta-analyses that reviewed 26 and 21 statin clinical trials, finding that statins were associated with a 33% and 29% relative risk reduction of myocardial infarction, respectively [29,30]. However, absolute risk reductions, which describe the magnitude of clinical effect size and are more practical for decision making in healthcare [31,32], were reported in the meta-analyses at much lower rates of reduced myocardial infarction, 0.89% and 1.3%. The meta-analyses also reported low absolute risk reductions for all-cause mortality and stroke from statin use, but limitations in analyses of pooled absolute risk reductions were noted due to heterogeneity of the trials [30].

Historically, biostatistician Jerome Cornfield introduced relative measures in clinical research in the 1950s while researching lung cancer and tobacco at the U.S. National Cancer Institute [33]. Cornfield pointed out that each of the absolute and relative measures serves a purpose and he proposed that the relative measure is useful to evaluate “the possible noncausal nature of an agent having an apparent effect.” Cornfield also stated that “the absolute measure would be important in appraising the public health significance of an effect known to be causal” [34], such as in clinical trials, the gold standard to measure causation or efficacy under controlled conditions through participant randomization to assigned groups [35]. Relative measures are more useful for uncontrolled observational studies that are designed to infer associations rather than causation, such as cohort studies, cross sectional studies, and case studies [36].

Risk in a clinical trial is the percentage of people in a group who have an incident of an event (disease, symptom, etc.), while the relative risk is simply a ratio of risks between groups. Importantly, a risk has a dimension, such as the number of events relative to the group size (e.g., the number of events per 1000 people). But a clinical practitioner has no way of knowing the dimension or magnitude of the effect size based solely on a risk ratio, which has no dimensions [37]. On the other hand, the risk difference or absolute risk reduction is a true measure of the effect size based on the baseline risk of the control group. By contrast, the relative risk reduction is simply the risk ratio subtracted from the null value of 1 (the null value has equal risks in groups). Importantly, the relative risk reduction is a ratio reduction, not a risk reduction per se. Unfortunately, compared to relative risk reductions, absolute risk reduction measures in clinical trials are often overlooked because they have a lower effect size [37]. Yet, absolute measures are essential for clinical research, and some researchers have proposed eliminating relative risk measures from clinical trials [38]. The United States Food and Drug Administration (FDA) and the U.S. Department of Health & Human Services (HHS) advises that both relative and absolute measures should be reported when communicating treatment risks and benefits to the public [39].

An example of a useful clinical measure to compare treatment efficacies is the reciprocal of the absolute risk reduction—the number needed to treat to prevent an event in one patient. The higher the number needed to treat, the less efficacious the treatment. For example, a cohort analysis based on major agency guidelines for statin use in the primary prevention of atherosclerotic cardiovascular disease investigated the number needed to treat. The analysis found that approximately 30 patients need to be treated with moderate-intensity statin therapy for 10 years to prevent a single atherosclerotic cardiovascular disease event in one patient (approximately 3% absolute risk reduction). The number needed to treat reduces to approximately 20 patients receiving high-intensity statin therapy over 10 years (5% absolute risk reduction) [40]. Guidelines included in the study were from the Canadian Cardiovascular Society, American College of Cardiology/American Heart Association, National Institute for Health and Care Excellence, US Preventive Services Task Force, and the European Society of Cardiology/European Atherosclerosis Society. Additionally, treatment goals of moderate-intensity statin therapy and high-intensity statin therapy aim to reduce baseline serum levels of LDL-C by 40% and 50%, respectively [41]. 

Table 1 summarizes calculations for relative and absolute risk reductions and number needed to treat in a clinical trial.

### Reassessing Landmark Statin Trial Findings

The landmark Scandinavian Simvastatin Survival Study in 1994, the first statin prospective randomized trial, accelerated acceptance of statin therapy into clinical practice, and “the results were remarkable” [42]. Reported findings of the study involving 4444 participants who were randomly assigned to simvastatin or placebo include a 30% relative risk reduction in all-cause mortality in the simvastatin group [43]. However, with 12% of deaths in the placebo group compared to 8% of deaths in the simvastatin group after 5.4 years, the unreported absolute risk reduction in all-cause mortality was much less remarkable at 4%. Consistently large differences in relative and absolute risk reductions of cardiovascular endpoints reported in statin trials imply that the relative benefits of statins communicated to the public often obscure the low absolute clinical efficacy of statin therapy. For example, the 2002 Medical Research Council and British Heart Foundation Heart Protection Study for simvastatin reported an 18% relative risk reduction of coronary mortality (5.7% risk with simvastatin versus 6.9% risk with placebo) [44]. But the study’s unreported absolute risk reduction of coronary mortality was just 1.2%. 

Moreover, in 2008, the Justification for the Use of Statins in Prevention: an Intervention Trial Evaluating Rosuvastatin (JUPITER) reported a relative risk reduction in fatal and non-fatal myocardial infarctions of 54% (hazard ratio: 0.46; 95% CI: 0.30–0.70) [45]. However, the unreported absolute risk reduction was only 0.41% (0.35% risk with rosuvastatin versus 0.76% risk with placebo). More recently, a 2019 prospective cohort study representing the general population of the United Kingdom found that over half of patients starting statin therapy did not lower LDL-C to optimal levels within two years [46]. Additionally, in a 2019 systematic review and meta-analysis of randomized trials, Yebyo et al. found that statins as a class reduced the relative risk of non-fatal myocardial infarction by 38%. But baseline risks and absolute risk reductions were unreported in the analysis, with the notable exception of absolute risks of myopathy and renal dysfunction which increased by 13% and 16%, respectively [47].

Misleading reports of relative risk reductions in landmark statin clinical trials have led health agencies to recommend aggressive lowering of LDL-C with statin therapy, and current global guidelines recommend statin therapy for all patients with atherosclerotic cardiovascular disease [42]. But statin therapy is not as efficacious as indicated by relative risk reductions in clinical trials, and small absolute risk reductions lack clinical significance. In contrast with statistical significance, clinical significance is based on the practicality of a treatment to improve the patient’s symptoms, health, and/or disease diagnosis in a way that impacts the course of the illness and the quality of life [48]. Furthermore, researchers question whether lowering LDL-C to normal levels reduces risk of atherosclerosis [49,50], and a causative relationship between LDL-C and atherosclerotic cardiovascular disease has not been proven [51]. Other mediating factors may play a causative role in the association of LDL-C with atherosclerotic disease which statins may not mitigate. Potential pathogenic factors of atherosclerosis that are under-investigated include cholesterol oxidation products, also known as oxysterols, which are discussed next.

## 4. Cholesterol and Oxysterols

The following is a summary based on a review of cholesterol, oxysterols, and atherosclerosis [52]. Ever since pathologist Rudolf Virchow first described atheroma as a swelling of the arteries in 1858 [53], researchers have advanced scientific knowledge of atherosclerosis. Atherosclerotic mechanisms include a link to hyperlipidemia in 1976 through the response-to-injury hypothesis by Ross and Harker [54], and foam cell formation from monocytes noted by Gerrity in 1981 [55]. Of relevance, the intima or inner layer of the blood vessel wall is lined with endothelial cells that come into direct contact with blood [56]. Fatty streaks begin to form in the intima in early atherosclerosis as LDL-C and white blood cells (leukocytes) penetrate endothelial cell membranes [57]. 

Although fatal thrombi from ruptures of atherosclerotic plaque were proposed by Falk in 1983 [58], this hypothesis was criticized more recently by Libby et al. based on infrequent ruptures of thin-capped fibroatheromas that rarely cause cardiovascular events [59]. Other findings of atherosclerotic mechanisms include the remodeled and enlarged vascular wall noted by Glagov et al. in 1985 [60]. Associations of high serum levels of LDL-C with high saturated fat intake, from the Seven Countries Study by Keys, supported the cholesterol hypothesis of atherosclerosis pathology [61,62]. Furthermore, a Nobel Prize was awarded to Brown and Goldstein in 1985 for their research on cholesterol homeostasis [63], on which statin therapy is based [64]. Still, the cause and prevention of atherosclerosis has not been established.

In addition to a dietary pattern high in saturated fat and cholesterol, evidence suggests that increased risk of atherosclerosis is associated with cholesterol oxidation products known as oxysterols [52]. Oxysterols form in food as dietary cholesterol is oxidized through food storage, processing, and preparation [65], which changes the polarity and structure of cholesterol molecules. This change in cholesterol prevents ingested oxysterols from properly packing into the phospholipid bilayer of the arterial vessel wall, as do normal cholesterol molecules [66]. In this oxidized state, and under conditions of lower shear stress when blood flow rates are reduced in bifurcated vessels, packing defects cause the endothelial cell membranes of the vessel to become more permeable to serum protein [67]. Specifically, the density of cholesterol and phospholipids in the membrane is reduced as large spaces form between the hydrophilic groups of the oxidized cholesterol, which alters cell membrane binding to serum proteins [67,68]. Consequently, increased amounts of serum LDL-C may enter the subendothelial space of the vessel wall, causing immune cells to oxidize the permeating LDL-C, forming foam cells and an atheroma which eventually blocks the vessel lumen [69]. Smaller serum lipoproteins permeate more easily through endothelial cell membranes affected by oxysterol-induced packing defects. 

**Figure 1:**
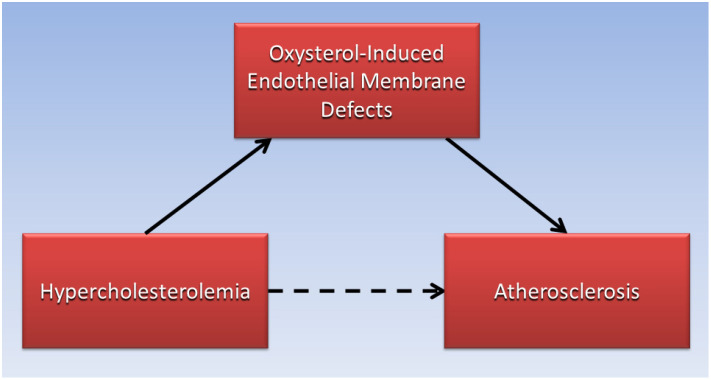
Endothelial membrane defects induced by oxysterols mediate the association of hypercholesterolemia with atherosclerosis.

Judging by the low absolute risk reductions of statins to reduce cardiovascular disease risk in clinical trials, reducing serum LDL-C levels with lipid-lowering drugs may not be sufficient to prevent LDL-C from permeating defective endothelial cell membranes and forming an atheroma. By contrast, high serum levels of oxysterols which cause endothelial membrane defects leading to increased permeability of LDL-C, have been associated with increased risk for atherosclerosis and cardiovascular disease in patients. For example, high levels of oxysterols were found in the plasma of patients having greater than 75% stenosis in their coronary arteries [70]. Additionally, a prospective cohort study found that higher plasma levels of the oxysterol 7-ketocholesterol was associated with a 36% increased hazard ratio of cardiovascular events and mortality over a median of 4.6 years in 1016 patients with coronary artery disease [71]. As illustrated in the directed acyclic graph (Figure 1), oxysterol-induced endothelial membrane defects may be the causative factor (solid arrows) that mediates the association (dotted arrow) of hypercholesterolemia with atherosclerosis, and more research is needed in this area.

Foods of animal origin undergo maximum formation of cholesterol oxidation products when thermally treated at cooking temperatures greater than 150 °C (302 °F) [72], and exposure to air and light in these foods during storage also increase levels of cholesterol oxidation products [73]. Importantly, foods of animal origin are proscribed in vegan diets, and restriction of food-derived oxysterols may account for the anti-atherosclerotic properties of plant-based dietary patterns [74]. For example, a vegan diet rapidly eliminated chest pain in angina cases reported by Ellis and Sanders in 1977 [75]. The first clinical trial demonstrating coronary heart disease reversal in patients following a low-fat vegetarian diet was conducted by Ornish et al. in 1990 [76].

## 5. Statins and Increased Cancer Risk

The low efficacy of statins to reduce absolute risks of cardiovascular disease implies that statins may be more useful as repurposed chemotherapy agents to treat cancer. Nevertheless, evidence from earlier clinical trials and animal studies suggest that statins are also carcinogenic [77,78]. A 1996 review of lipid-lowering drugs by Newman and Hulley found that statins are carcinogenic in rodents, with some exposures approaching prescriptions for humans, although statin carcinogenicity in human clinical trials at the time were considered inconclusive [79]. That same year, the Cholesterol and Recurrent Events (CARE) clinical trial reported adverse events in patients with myocardial infarction who were randomized to pravastatin or placebo. Among 286 women in the pravastatin group, 12 had developed breast cancer by follow-up compared to 1 woman with breast cancer among 290 women in the placebo group. Pravastatin raised the risk of breast cancer to 4.20%, an absolute risk increase of 3.86% compared to the placebo baseline risk of 0.34%, *p* = 0.002 [80], and a relative risk increase of 11%. 

The Prospective Study of Pravastatin in the Elderly at Risk (PROSPER) randomized adults aged 70 to 82 years to pravastatin (*n* = 2891) or placebo (*n* = 2913). In a 2002 report, new cancer diagnoses at 3.2 years follow-up in the pravastatin group (245 cancers) compared to placebo (199 cancers) raised the risk of breast cancer to 8.47%. This was an absolute risk increase of 1.64% in the pravastatin group compared to the placebo baseline risk of 6.83%, *p* = 0.02) [81], and a relative risk increase of 24%. In 2008, the Simvastatin and Ezetimibe in Aortic Stenosis (SEAS) clinical trial that randomized patients to simvastatin-ezetimibe (*n* = 944) or placebo (*n* = 929) reported cancer incidence from simvastatin-ezetimibe (105 cancers) and placebo (70 cancers). Simvastatin-ezetimibe raised the risk of cancer to 11.12%, an absolute risk increase of 3.59% above the placebo baseline risk of 7.53%, *p* = 0.01) [82], and a relative risk increase of 48%. 

Although these examples show modest increases in the absolute risk of cancer from use of statins in randomized controlled trials, these findings suggest a carcinogenic effect from statin use. In population studies, a 2004 US case-control study using the General Practice Research Database found increased odds ratios of 3.5 for colon cancer (95% CI: 1.1–10.9) and 4.2 for rectal cancer (95% CI: 1.0–16.6) in current statin users of five years or longer [83]. In 2008, another US case-control study found that obese men had 80% increased odds of prostate cancer (95% CI: 1.1–3.0) associated with use of statins for five years or longer [84]. A series of nested case-control studies conducted in the United Kingdom in 2011 found that use of statins for more than four years was associated with significantly increased odds of 29% for bladder cancer (95% CI: 1.08–1.54). The studies also found a 23% increased odds for colorectal cancer (95% CI: 1.10–1.38) and 18% increased odds for lung cancer (95% CI: 1.05–1.34) [85]. In 2011, a case-control study examining 388 prostate cancer cases in the Taiwan population found that increasing odds of prostate cancer were associated with an increasing cumulative dose of statin use, with an increase of 89% odds at the highest cumulative dose (95% CI: 1.03–3.37) [86]. 

Additionally, a 2013 case-control study of women who used lipophilic statins for ten years found that odds of breast cancer were 74% greater for invasive ductal carcinoma (95% CI: 1.05–2.86) and 68% greater for invasive lobular carcinoma (95% CI: 1.02–2.76) [87] compared to never users. An analysis of statin use associated with cancer in 2015 in Japan found increased odds ratios of 1.29 for colorectal cancer (95% CI: 1.20–1.38), 1.35 for pancreatic cancer (95% CI: 1.24–1.47), and 1.25 for prostate cancer (95% CI: 1.17–1.34) [23]. More recently, a 2018 analysis of data collected from 763 postmenopausal women in the Women’s Health Initiative found a 30% increased risk of ovarian cancer (95% CI: 1.04–1.62) associated mostly with pravastatin use [88].

## 6. Statins’ Anticancer Effect and Confounding by Indication

Similar to other researchers, Mamtani et al. found that statin use was associated with decreased prevalence of colorectal cancer in a case-control study—odds of 0.95 (95% CI: 0.91–0.99) and 0.92 (95% CI: 0.85–0.99) in long-term and short-term users of statins, respectively, compared to nonusers [89]. However, Mamtani et al. also found that the association of cancer risk in a subgroup of cases who discontinued statin therapy showed no difference compared to cases who continued statin therapy. These findings inferred that statin therapy made no difference in cancer risk, and prompted the researchers to suggest that confounding by indication had biased the study findings.

Confounding by indication is defined as “a distortion that modifies an association between an exposure and an outcome, caused by the presence of an indication for the exposure that is the true cause of the outcome” [90]. For example, an outcome attributed to a treatment (such as lower cancer risk attributed to statin therapy) is instead caused by or related to the condition indicating the need for the treatment (high serum cholesterol levels). In this case, statins are indicated to treat hypercholesterolemia and prevent atherosclerosis and other cardiovascular conditions, implying that hypercholesterolemia and atherosclerosis are somehow related to reduced cancer risk.

Importantly, most researchers investigating the statin-cancer controversy do not appear to be aware of an inverse relationship between the incidence of atherosclerosis and cancer, nor have researchers considered how this relationship could confound outcomes in statin studies. An inverse relationship between atherosclerosis and cancer has been reported in the research literature for over a century, long before the introduction of statins. But no satisfactory explanation has been established to account for this observation. A more detailed discussion of the inverse association of cancer and atherosclerosis is provided elsewhere [91] and a few key studies are briefly summarized here.

Pathologists of the early 20th Century described the low prevalence of atherosclerosis in cases of cancer [92], and a more recent 2015 study of 5262 elderly adults found that mortality from cancer was reduced between 30% and 40% in adults who had died with atherosclerotic disease [93]. A 2016 study examining over 2300 autopsy records revealed that various cancers were inversely correlated with atherosclerosis [94]. A significant inverse correlation between atherosclerosis and cancer was also confirmed by Li et al. in an analysis of over 1000 autopsy reports and a review of data from the Harvard Catalyst Shared Health Research Information Network [95]. 

The following section introduces phosphate toxicity as a potential mediating factor proposed to explain the inverse association of cancer with atherosclerosis and further explain confounding by indication in statin trials finding reduced cancer risk.

## 7. Tumorigenesis and Phosphate Toxicity

A detailed review of tumorigenesis and phosphate toxicity is available elsewhere [96]; this section summarizes key findings. As an essential micronutrient, ingested dietary phosphorus in the form of inorganic phosphate (Pi) is systemically regulated through a network of endocrine hormones from the kidneys, bone, intestines, and parathyroid glands [27]. Dysregulation of this network can cause the accumulation of extracellular and intracellular Pi, producing a condition known as phosphate toxicity that is harmful to most major organ systems. Studies show that elevated Pi accumulates in the extracellular matrix of the tumor microenvironment [97]. For example, increased genetic expression of transporters that cross cancer cell membranes facilitate greater intracellular phosphate absorption from the extracellular matrix of the tumor microenvironment, enabling sequestration of excessive amounts of Pi into cancer cells. Furthermore, compared to genetic expression in normal tissue, the sodium phosphate cotransporter NaPi2b (encoded by the SLC34A2 gene) is overexpressed in cancer cells of the breast, lung, and thyroid [98], and NaPi2b overexpression was also found in ovarian carcinomas [99]. Levels of Pi measured in cancer cells of the lung and colon can contain up to twice the amount of Pi compared to Pi levels in normal cells in these organs [100]. 

Bobko et al. conducted an in vivo assessment of Pi in the interstitial microenvironment of tumors using advanced electron paramagnetic resonance profiling, and confirmed a two-fold increase in extracellular Pi concentrations linked to tumor metastasis [101]. In a 1954 study, Ward et al. described how biogenesis of nuclear ribonucleic acid (messenger RNA, transfer RNA, and ribosomal RNA) is increased by excessive Pi uptake, which promotes growth of cancer cells through increased protein synthesis [102]. The authors also described experiments in which suppression of phosphorus uptake by nuclear RNA delayed carcinogenesis in precancerous tissue. Another study found that high concentrations of Pi in the microenvironment of breast cancer cells and lung cancer cells stimulated angiogenesis and neovascularization, which are important for tumor growth [103].

Additionally, high levels of dietary phosphate increased lung cancer in lab mice [104]. Excess Pi increased cancer cell proliferation in the mice by activating cell signaling pathways involving phosphoinositide 3-kinase, protein kinase B (Akt), and mechanistic target of rapamycin (mTOR) kinase. The researchers also found that Pi suppressed cell apoptosis by deactivating phosphatase and tensin homolog (PTEN), a tumor suppressor, and Pi deactivated carboxyl-terminal modulator protein (CTMP), which downregulates activity of Akt [105]. Regulation of tumor growth through the GTP-binding protein N-ras signaling pathway was also activated by a diet high in phosphate fed to mice, which increased skin cancer (papilloma) in the mice by approximately 50% [106].

After 24 years of follow up, risk of high-grade and lethal prostate cancer was associated with high intake of dietary phosphate in the Health Professionals Follow-Up Study [107]. According to an analysis of over 34,000 adults in the U.S. National Health and Nutrition and Examination Survey (NHANES) for the years 2001–2014, the highest percentage of dietary phosphate in the diet of Americans was supplied by grains, meats, and dairy products [108]. Additionally, a Swedish study found a 44% increased risk of cancer mortality in women associated with three or more glasses of milk consumed daily compared to less than one glass [109].

Compared to patients without cancer, researchers found that some cancer patients had more than twice the mean level of serum phosphate: 7.80 (±2.24) mg/dL and 3.38 (±0.58) mg/dL, respectively (*p* < 0.001) [110]. An analysis of cohort data from the Swedish AMORIS study found a positive association between high serum phosphate levels and risks for cancers of the pancreas, lung, thyroid, and bone in men, and cancers of the lung, esophagus, and nonmelanoma skin cancer in women [111]. Although the researchers found that high serum phosphate levels were negatively correlated with breast cancer and endometrial cancer risk, this could be related to rapid cell division in female reproductive tissue that requires higher amounts of phosphate. Other studies found that higher serum phosphate in patients diagnosed with non-small cell lung cancer and small cell lung cancer was predictive of the stage of disease and patient survival [112]. Patient survival also was negatively associated with hyperphosphatemia in a cohort of over 1200 patients with colorectal cancer [113]. Furthermore, based on the growth rate hypothesis, a mathematical model of tumor dynamics by Kuang et al. predicted that lowering a tumor’s uptake of phosphorus by 50% will reduce the tumor’s size by 75% [114].

Inorganic polyphosphate (polyP), a polymer of single phosphate groups (orthophosphates) linked by phosphoanhydride bonds, has been proposed as a stored energy source for cellular proliferation in tumorigenesis, and polyP is exceedingly concentrated in several types of cancer cells [115]. Lowering levels of polyP alters the morphology and viability of cancer cells. Importantly, polyP does not require oxygen to be directly converted into adenosine triphosphate for energy, which eliminates tumor dependency on intermediate steps like anaerobic glycolysis proposed by the Warburg effect [116].

Ectonucleotide pyrophosphatase/phosphodiesterase 1 (ENPP1) hydrolyzes adenosine triphosphate to produce pyrophosphate (two phosphate groups linked to oxygen through phosphodiester bonds) which inhibits the deposition of hydroxyapatite in skeletal tissue during bone remodeling [117]. ENPP1 is associated with cancer as well as with diabetes, cardiovascular disease, and osteoarthritis—diseases linked to statins through potential mediation by phosphate toxicity, discussed in Section 9. An elevated level of ENPP1 in human primary breast tumors is a potential mediating factor in the metastasis of breast cancer to bone [118].

A recent 2024 review by the present author supports the potential role of reduced dietary phosphate load associated with spontaneous regression of tumors, which shrinks tumor size without conventional therapy [119]. Tumor reversion, which returns cancer cells to normal cells when cancer cells are transplanted into a normal microenvironment, may also be associated with reduced dietary phosphate load, and further research is needed in this area. The following section discusses the effect of phosphate in dietary patterns.

## 8. Dietary Patterns and Confounding by Indication

Increased risk of cancer is associated with a dietary pattern that is high in phosphorus [96], and high dietary phosphorus is correlated with high dietary protein as well. Generally, a mixed diet has approximately 12–14 mg of phosphorus for each gram of protein [120]. By comparison, food items with more dietary fat generally contain less phosphorus, e.g., cooking oils often contain no phosphorus, and the level of phosphorus in butter is over five times lower than phosphorus in nonfat milk [121]. Therefore, dietary patterns in which high-fat and high-cholesterol foods provide most of the daily caloric intake tend to be potentially more atherogenic and less tumorigenic. 

Some people may have a greater risk for either atherosclerosis or cancer depending on which type of dietary pattern predominates in their lifestyle: either an atherogenic pattern containing more dietary cholesterol and oxysterols or a tumorigenic pattern containing more protein and phosphate [91]. Accordingly, people more inclined to follow a high dietary cholesterol pattern are more likely to have higher serum cholesterol levels, indicating a need for statin therapy to reduce risk of atherosclerosis and other cardiovascular conditions. However, due to confounding by indication, the true cause of lower cancer risk associated with statin use is potentially cancer’s inverse relationship with atherosclerosis. More research is needed to investigate the correlation of atherogenic and tumorigenic dietary patterns with the inverse relationship between cancer and atherosclerosis. Furthermore, effects of atherogenic and tumorigenic dietary patterns do not preclude the effects of diets that combine both patterns, i.e., dietary patterns with high intake of saturated fat, cholesterol, protein, and phosphate, and research is needed to investigate the pathogenic effects of various combinations of dietary patterns in cancer and cardiovascular disease.

Adjusting data analyses in statin studies to control confounding by indication due to the inverse relationship between cancer and atherosclerosis could negate much of the anticancer effect attributed to statins. For example, in 2015, participants in a population-based prospective study in Spain who had symptomatic atherosclerotic disease were found to have a 30–40% lower rate of cancer mortality compared to participants without symptomatic atherosclerotic disease [93]. A German study found low prevalence of atherosclerosis in deceased cancer patients (odds ratio of generally 0.5 or less in specific cancer types) [94], and studies in the 1950s found low atherosclerosis risk in cancer [92,122,123]. These earlier findings preclude the possibility that the inverse association between cancer and atherosclerosis was caused by the introduction of statins in 1987 [124]. Future studies are needed to confirm the effect of confounding by indication for statin therapy associated with reduced cancer risk.

## 9. Statin-Induced Rhabdomyolysis and Phosphate Toxicity

Rhabdomyolysis is the breakdown of muscle tissue caused by physical trauma, but drugs, toxic agents, and infections are nontraumatic factors that appear at least five times more common in causing rhabdomyolysis [125]. Cabral et al. recently suggested that statins are the leading cause of drug-induced rhabdomyolysis, which damages striated muscle fiber and produces symptoms of myalgia and muscle weakness [126]. Clinical effects from the release of muscle components in rhabdomyolysis also include dark urine from myoglobinuria and a rapid increase in serum creatine kinase levels [127]. Additionally, Cabral et al. noted that damaged muscle cells release phosphorus and potassium causing hyperphosphatemia and hyperkalemia. Implicitly, findings of carcinogenicity associated with statin use may be caused indirectly by statin-induced rhabdomyolysis which gradually stimulates tumor growth through increased circulation of released Pi. 

Cancer treatments like chemotherapy and radiation can kill many cancer cells which may lead to tumor lysis syndrome as excessive amounts of intracellular components are released into the serum, causing hyperphosphatemia [128]. Of relevance, rhabdomyolysis was noted to affect serum levels in a manner similar to tumor lysis syndrome [129]. Additionally, secondary sarcomas occur less often in patients treated with surgery alone compared to surgery with chemotherapy and radiation [130], possibly by avoiding tumor lysis syndrome as the tumor and its intracellular contents are excised during surgery.

Statin therapy-induced rhabdomyolysis, although rare, can be fatal and is thought to be caused by reduced levels of ubiquinone, coenzyme Q, which is normally produced in the HMG-CoA pathway [131]. Ubiquinone is necessary to facilitate electron transport in the mitochondrial respiratory chain, and lack of ubiquinone can disrupt the production of cellular energy, leading to death of skeletal muscle cells [132] and release of muscle-cell components into the blood stream [133].

Hypercholesterolemia treated by statins is defined as levels of plasma cholesterol that exceed 200 mg/dL for total cholesterol and 100 mg/dL for LDL-C [134]. The lipid-lowering effects of statins occur by several mechanisms. Statins reduce cholesterol synthesis by lowering the conversion of sterol precursors to cholesterol in the mevalonate pathway [135], and statins lower plasma levels of very-low density lipoproteins and intermediate-density lipoproteins [136]. Statins most commonly used by practitioners currently include simvastatin, rosuvastatin, pravastatin, and atorvastatin [41]. Simvastatin and atorvastatin are lipophilic statins that more readily absorb into cells by passive transport through the phospholipid cell membranes. Lipophilic statins are associated with greater statin-associated muscular symptoms in comparison with hydrophilic statins like pravastatin and rosuvastatin which depend on active transport by transport proteins.

Simvastatin is highly sensitive to metabolization by the cytochrome p450-3A4 (CYP3A4) enzyme system, and CYP3A4 inhibitors, including some antibiotics, antifungals, protease inhibitors, and calcium channel blockers, raised the simvastatin serum concentration and induced rhabdomyolysis in a case study [137]. A 2002 analysis of data investigating six statins from the Adverse Event Reporting System of the FDA suggested that drug interactions caused 57% of statin-induced rhabdomyolysis cases [138]. Additionally, a 2012 United Kingdom study found that the interaction of a CYP3A4-metabolized inhibitor with a CYP3A4-metabolized-statin accounted for approximately 30% of patients with statin-induced rhabdomyolysis [139]. More recently, a 2023 analysis of data from the World Health Organization’s pharmacovigilance database found that the reporting odds ratio of rhabdomyolysis associated with simvastatin was 2.20 compared to six other statins [140]. More research is needed in drug interactions and statin-induced rhabdomyolysis. Furthermore, other adverse effects of statins may be mediated by phosphate toxicity associated with statin-induced rhabdomyolysis, as in the following comorbid conditions.

### 9.1. Diabetes

Diabetes mellitus is associated with statin use, however, the mechanism that increases diabetes risk remains unclear [141]. Diabetes is also associated with phosphate toxicity related to mitochondrial dysfunction from calcium-phosphate accumulation in the mitochondrial matrix of insulin-producing pancreatic beta cells [142]. Additionally, serious cases of rhabdomyolysis are not uncommon in diabetes [143], and future research should investigate the risk of diabetes related to statin-induced rhabdomyolysis, potentially mediated by phosphate toxicity.

### 9.2. Parkinson’s Disease

Although research findings of Parkinson’s disease risk associated with statin use are mixed, researchers from Pennsylvania State University recently found significantly increased risk of Parkinson’s disease in a large case-control study of 2322 cases [144]. Parkinson’s disease is also associated with phosphate toxicity related to mitochondrial dysfunction in the dopamine-producing cells of the substantia nigra [145]. Effects of dysregulated phosphate in Parkinson’s disease are associated with opening of the mitochondrial permeability transition pore, DNA damage, inflammation, increased reactive oxygen species, and cell death. Hyperphosphorylation of tau protein is also associated with α-synuclein and Lewy body dementia in Parkinson’s disease. Similar to diabetes, future research should investigate the risk of Parkinson’s disease related to statin-induced rhabdomyolysis, potentially mediated by phosphate toxicity.

### 9.3. Cardiomyopathy

In the 2008 report of the JUPITER trial, the statin group had more fatal myocardial infarctions compared to myocardial infarction fatalities in the placebo group (nine and six fatalities, respectively) [45]. Relatedly, cardiomyopathy is associated with long-term statin use, called statin-associated cardiomyopathy [146]. A case study reported myocarditis due to inflammation that occurred concurrently with statin-induced acute necrotizing myositis [147]. The researchers called for studies to establish acute myositis with myocarditis as a plausible adverse effect of statin therapy. Additionally, acute rhabdomyolysis is associated with high serum levels of inflammatory cytokines and chemokines [148], and future studies should investigate serum levels of inflammatory factors, including phosphate toxicity, that mediate the association of statin-induced rhabdomyolysis with myocarditis.

### 9.4. Kidney Disease

Both traumatic and nontraumatic rhabdomyolysis are associated with acute kidney injury [149]. A 2016 retrospective cohort study of 6342 statin users and 6342 matched nonusers of statins found that prolonged statin use (8.4 years) was associated with 30% increased odds of acute kidney injury (95% CI: 1.14–1.48) [150]. A subset of patients without comorbid conditions in the study found 53% increased odds of chronic kidney disease (95% CI: 1.27–1.85). Importantly, hyperphosphatemia and adverse renal effects from phosphate toxicity are well established in kidney disease [151], and kidney disease risk in statin users is potentially mediated by rhabdomyolysis-induced phosphate toxicity.

### 9.5. Bone Fracture, Cataract, and Coronary Artery Calcification

Findings are mixed on whether statins reduce risk of bone fractures [152,153]. Bone mineral disorders leading to fractures can be caused by hyperphosphatemia, which induces bone resorption mediated by secondary hyperparathyroidism [154]. As high serum levels of Pi combine with serum calcium in hyperphosphatemia, calcium serum levels fall below normal, triggering release of parathyroid hormone in secondary hyperparathyroidism which releases calcium from bone to restore serum calcium. Serum calcium-phosphate product formed in hyperphosphatemia can also lead to abnormal mineral deposition in bone—metastatic calcification and osteosclerosis [155]—and ectopic calcification in soft tissue [27]. Importantly, ectopic calcification related to dysregulated phosphate metabolism should be investigated in findings of a meta-analysis reporting an association of statins with higher bone mineral density. [156]. The findings did not include reductions in fracture risk which suggests abnormal bone overgrowth in osteosclerosis [157]. Ectopic calcification related to hyperphosphatemia can also lead to cataracts [158], and cataracts have been associated with statin use [159], but findings are controversial. Additionally, statin use is associated with coronary artery calcification [160,161], warranting further investigations of associations with dysregulated phosphate metabolism.

### 9.6. Vitamin D Deficiency

Approximately 40 years of research has failed to reduce cancer risk with vitamin D supplementation, likely because the association of vitamin D deficiency and cancer is mediated by dysregulated phosphate metabolism, common in chronic kidney disease (CKD) [162]. Specifically, vitamin D deficiency is severe in many patients with CKD, which is further impaired by reduced renal conversion of 25-(OH)vitamin D into bioactive calcitriol, 1,25 dihydroxy-vitamin D [163]. The kidneys lower calcitriol levels to reduce intestinal absorption of Pi. This Pi regulatory mechanism may also affect the association of vitamin D deficiency with statin use mediated by rhabdomyolysis-induced hyperphosphatemia. Myalgia, a symptom of statin use, is also a symptom of vitamin D deficiency, and a cross-sectional study of 287 statin users with muscle symptoms compared to 923 statin users without muscle symptoms found that insufficient and deficient vitamin D status (<30 nmol/L) was significantly associated with statin-associated muscle symptoms [164]. A 2017 systematic review and meta-analysis of studies on statin therapy found contradicting results of both higher and lower levels of vitamin D among statin users [165]. Contradicting results could be explained by confounding from indication for statin use, i.e., higher intake of dietary cholesterol could mediate higher vitamin D levels in statin users compared to higher phosphate intake that mediates lower vitamin D levels in statin users; more studies are needed in this area. Furthermore, a 2023 randomized controlled trial found no benefit from vitamin D supplementation for statin-induced muscle pain [166], adding strength to the evidence of an indirect relationship between statin-induced muscle symptoms and vitamin D levels, mediated by phosphate toxicity. Similar lack of benefits for statin-induced muscle pain from vitamin D supplementation was found in a larger 2022 randomized trial of 2083 new users of statins [167].

### 9.7. Periodontal Disease

Similar to dysregulated calcium metabolism in bone mineral disorders, periodontal disease with loss of alveolar bone and other tooth-supporting tissue is also associated with phosphate toxicity [168]. This relationship may explain the association of statin use with periodontal disease mediated by rhabdomyolysis-induced hyperphosphatemia. Some studies have found reductions in alveolar bone loss from topical application of statins compared to systemic delivery of the drugs [169]. Nevertheless, odds ratios of chronic periodontitis occurrence were higher in 169,381 statin users compared to non-user-matched controls in a large 2022 long-term prospective study [170]. The researchers noted that the underlying mechanism explaining the increased risk of periodontal disease associated with statin use is not clear, and more research is needed in this area.

### 9.8. Osteoarthritis

A 2022 systematic review and meta-analysis found that statin use is significantly associated with increased risk of osteoarthritis, especially at high doses [171]. Importantly, prevalence of osteoarthritis is high in people with CKD, and evidence suggests common risk factors are shared in the pathophysiology of osteoarthritis and CKD [172]. Shared risk factors may include dysregulated phosphate metabolism, which should be investigated as a potential mediating factor in investigations linking statins with osteoarthritis.

## 10. Summary of Statin-Cancer Risk Factors

Table 2 shows a brief summary of reviewed evidence from articles cited in the present paper, which are synthesized into new knowledge to fill in evidence gaps involving statins and cancer risk factors.

**Figure 2:**
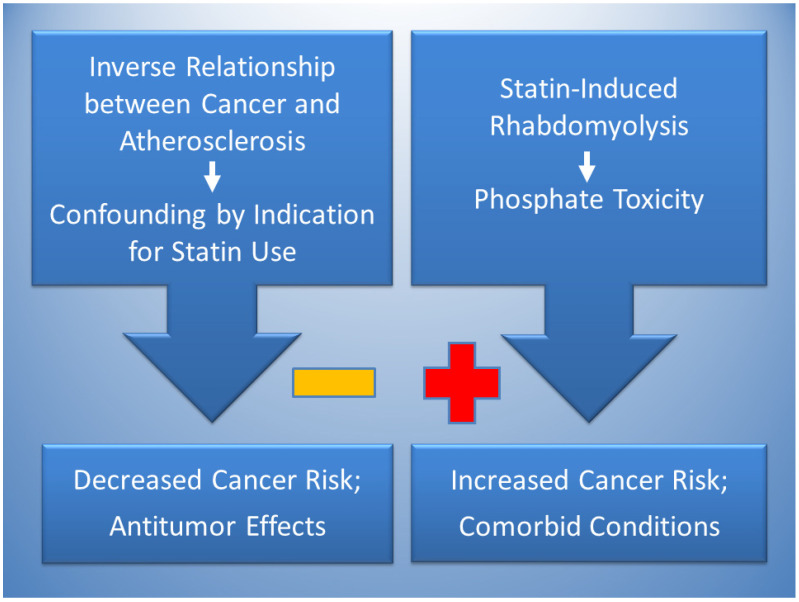
The inverse relationship between cancer and atherosclerosis, potentially mediated by low phosphate levels in atherogenic dietary patterns, is proposed to account for confounding by indication in the association of statins with decreased cancer risk (negative sign). Statins also have antitumor effects. Conversely, statin-induced rhabdomyolysis is proposed to increase cancer risk (positive sign) through phosphate toxicity. Phosphate toxicity is a mediating factor that potentially contributes to comorbid conditions associated with statin use, including diabetes, Parkinson’s Disease, cardiomyopathy, and renal disease.

Figure 2 proposes that the effects of statins can simultaneously lower and raise risks of cancer. Reduced cancer risks are likely due to confounding by indication in studies finding an association between statin use and cancer protection, which is potentially explained by the inverse relationship of atherosclerosis and cancer. Dietary patterns that are high in saturated fat and cholesterol tend to have atherogenic effects, in contrast with dietary patterns high in protein and phosphate, which tend to have tumorigenic effects [91]. Accordingly, a dietary pattern high in saturated fat and cholesterol that indicates use of statins to control hypercholesterolemia and prevent atherosclerosis may be generally lower in dietary phosphate which may reduce the risk of cancer, causing confounding by indication. Statin therapy also has antitumor effects related to cytotoxicity.

By contrast, evidence supports an association of statins with increased cancer risk involving rhabdomyolysis which releases Pi into the serum and is associated with increased tumorigenesis. Conditions comorbid with statin use should also be investigated for the common mediating factor of phosphate toxicity, including diabetes, kidney disease, Parkinson’s disease, cardiomyopathy, osteoarthritis, periodontitis, vitamin D deficiency and conditions associated with ectopic calcification such as bone fractures, cataracts, and coronary artery calcification.

## 11. Future Research

Suggestions for future research include the following: More reviews should examine the literature on statin clinical trials, past, present, and future, and report absolute risk reductions and number needed to treat to communicate the clinical significance of trial effect sizes to the public. Systematic reviews and meta-analyses of statin trials should also be reviewed and updated with absolute measures of absolute effect sizes. In addition, major agency guidelines for statin use to prevent atherosclerotic cardiovascular disease should be reevaluated in view of similar reporting problems that ignore absolute effect sizes in clinical trials.

In addition to focusing attention on hypercholesterolemia as a cause of atherosclerosis, research should focus attention on other causative factors such as effects of dietary oxysterols. Alternative treatments to statins for preventing atherosclerosis should also be investigated, such as clinical testing of plant-based diets that are low in dietary cholesterol. Furthermore, analyses of oxysterol content in common foods should be made widely available to the public, and cholesterol education programs should be updated on cardiovascular risks associated with dietary oxysterol intake.

Clinical research on statins as cytotoxic agents for cancer treatments can also be further pursued. However, more emphasis should be placed on cancer prevention through lifestyle modifications of dietary phosphate intake. For example, low-phosphate diets are already used by hemodialysis patients to control hyperphosphatemia [173], and clinical research should test similar low-phosphate diets in patients with cancer [174]. Finally, murine tumor models can be used to test effects on tumorigenesis associated with Pi released during statin-induced rhabdomyolysis [175]. Animal models of atherosclerosis can also test effects on atherosclerosis associated with atherogenic and tumorigenic dietary patterns containing inverse levels of dietary cholesterol and phosphate [176].

## 12. Conclusions

The solution to the controversy surrounding statins and cancer appears to be multifactorial, involving bias and pathophysiological mechanisms, some of which are mediated by phosphate toxicity. The association of statin use with decreased cancer risk appears to involve confounding by indication due to the inverse relationship between atherosclerosis and cancer. Furthermore, statin trials often report relative risk reductions without absolute risk reductions, biasing risk-benefit analyses in clinical decisions to prescribe statin therapy. Oxysterols potentially mediate the association of hypercholesterolemia with atherosclerosis and further research is needed in this area. Although cytotoxicity from statins has antitumor effects, statins are also associated with increased cancer risk potentially involving statin-induced rhabdomyolysis leading to hyperphosphatemia, phosphate toxicity, and tumorigenesis. The mediating role of phosphate toxicity in statin-induced rhabdomyolysis may also explain conditions that are comorbid with statin use. More research is warranted to investigate the mechanisms linking statins with the cause and prevention of cancer.

## Figures and Tables

**Table 1:** Relative and Absolute Risk Reductions and Number Needed to Treat.

**Risk**	The percentage of an event/outcome/endpoint in a clinical trial that occurs in each of two groups, the experimental group receiving a treatment/intervention and the control group receiving a placebo.
**Relative Risk**	The ratio of the experimental group risk relative to (divided by) the control group risk (the baseline risk).
**Relative Risk Reduction**	The relative risk subtracted from the null value of 1.00. The null value has equal risks in both groups.
**Absolute Risk Reduction**	The experimental group risk subtracted from the control group risk, indicating the size of the risk difference.
**Relative Risk Reduction** **(alternative calculation)**	The absolute risk reduction is divided by the baseline risk.
**Number Needed to Treat**	The null value (1.00) divided by the absolute risk reduction (i.e., the reciprocal of the absolute risk reduction) which indicates the number of treated patients needed to reduce one event.

**Table 2:** Brief Summary of Reviewed Evidence and Knowledge Synthesis.

Reviewed Evidence	Knowledge Synthesis
Statin therapy is associated with cancer protection [6,7], plausibly due to confounding by indication for statin therapy [89].	Confounding by indication for statin therapy is potentially caused by an inverse relationship between cancer and atherosclerosis [91].
Statin therapy is associated with increased cancer risk [79,87,88]. Rhabdomyolysis is also an adverse effect of statin therapy which breaks down skeletal muscle [126].	Phosphate released into serum from rhabdomyolysis increases the risk of phosphate toxicity and subsequent increased risk of tumorigenesis [100,101].
High relative risk reductions of cardiovascular disease are reported in clinical trials of statin therapies [28,42,45]. But low absolute risk reductions and low clinical effect sizes of statin therapy are often unreported in clinical trials [29,30].	The U.S. FDA and HHS advise reporting both relative and absolute risk reductions in clinical trials [39], which suggests that the putative benefits of statin therapy may not outweigh adverse effects.
Statin use is strongly associated with comorbidities: e.g., diabetes [141], Parkinson’s disease [144], cardiomyopathy [146], and kidney injury [150].	Phosphate toxicity and related rhabdomyolysis potentially mediate the association of statins with comorbidities: e.g., diabetes [142], Parkinson’s disease [144], inflammation [148], and CKD [151].

## Data Availability

No new data were created.
